# Progression of myopia among undergraduate students of Fatima Jinnah Medical University, Lahore

**DOI:** 10.12669/pjms.42.9.16073

**Published:** 2026-09

**Authors:** Huma Kayani Saigol, Maham Javed, Hamza Riaz, Abdul Rahman Alvi

**Affiliations:** 1 Huma Kayani Saigol, FCPS, FRCS, VRO, MHPE Principal, Khawaja Muhammad Safdar Medical College, Sialkot, Pakistan; 2 Maham Javed, MBBS, FCPS VRO Fellowship, Department of Ophthalmology, Sir Ganga Ram Hospital, Lahore Pakistan; 3 Hamza Riaz, MBBS Ex Registrar, Dept. of Radiology, Sir Ganga Ram Hospital (SGRH), Sir Ganga Ram Hospital, Lahore Pakistan; 4 Abdul Rahman Alvi, MS Information Security Deputy Director IT, Fatima Jinnah Medical University, Lahore, Pakistan

**Keywords:** Behavior, Degenerative, Myopia, Myopia, Medical, Ocular, Refraction, Students

## Abstract

**Background and Objective:**

The global rise in myopia has become a significant public health concern, with a projected prevalence affecting nearly half of the world’s population by 2050. Despite increasing prevalence in low- and middle-income countries (LMICs), longitudinal data on myopia progression, especially in high-risk academic cohorts, remain limited. This study investigates the five-year progression of myopia and its behavioral correlates among undergraduate female medical students in Pakistan.

**Methodology:**

A longitudinal prospective study was conducted at Fatima Jinnah Medical University, Lahore, including 304 students enrolled in the 2020 MBBS batch. Annual measurements of spherical equivalent refraction (SER), self-reported screen time, and study time were collected over five academic years, from January 2020 to December 2025. Descriptive statistics summarized SER progression and behavioral exposure. Linear mixed-effects models (LMM) assessed SER changes across time. Multivariable linear regression evaluated predictors of SER progression (ΔSER). Spearman’s correlation and subgroup analyses examined associations with behavioral and demographic variables.

**Results:**

Of the 304 students, 48 had complete longitudinal SER data. The mean SER progressed from –1.66 D (SD ±2.41) at baseline to –2.73 D (SD ±1.78) after five years, with a mean ΔSER of –1.07 D (SD ±2.85). Baseline SER was a significant predictor of progression (β = –0.96; 95% CI: –1.20 to –0.73; p < 0.001). No significant associations were found between ΔSER and screen or study time. Subgroup analysis showed no difference in progression by family history of myopia.

**Conclusion:**

This study adds to the existing Pakistani literature on myopia among medical students by providing longitudinal data on refractive change over a period of five-year. Findings highlight baseline refractive error as the strongest determinant of progression, emphasizing the need for early detection and targeted surveillance in LMIC academic populations.

## INTRODUCTION

Myopia, or nearsightedness, is a global public health concern with rapidly increasing prevalence, particularly among younger populations. It is estimated that by 2050, nearly 50% of the world’s population, i.e., approximately five billion people will be affected by myopia, with up to one billion individuals experiencing high myopia and its associated vision-threatening complications, including retinal detachment, myopic macular degeneration, and glaucoma.^1^ The rapid rise in prevalence, especially in East and Southeast Asia where myopia affects 80–90% of teenagers, reflects not only a genetic predisposition but also profound environmental shifts in behavior, education, and lifestyle.^2^ In contrast, prevalence in European populations ranges from 17% to 36%, reflecting regional differences in educational intensity and outdoor activity exposure.^3^ This alarming trajectory has led to myopia being classified as a global epidemic by the World Health Organization.^4^ Without immediate public health interventions, the rising burden of high myopia threatens to overwhelm healthcare systems, particularly in low- and middle-income countries.

Myopia onset refers to the initial development of refractive error in childhood or adolescence, typically marked by the shift of the eye’s axial length and refractive power toward negative diopters.^1^ In contrast, myopia progression denotes the subsequent worsening of this refractive error over time, often quantified by a change in spherical equivalent refraction (SER), typically ≥0.50 diopters (D) over one year.^5^ If left unaddressed, progressive myopia can lead to irreversible structural changes in the retina and sclera, culminating in visual impairment and blindness. Complications such as posterior staphyloma, choroidal neovascularization, and retinal detachment are increasingly common in individuals with high myopia.^6^ Understanding and mitigating this progression is crucial, as it increases the likelihood of severe ocular pathology later in life.

Multiple studies have established that both genetic and environmental factors contribute significantly to the development and progression of myopia. Genetic contributions are supported by twin and familial aggregation studies, as well as genome-wide association studies identifying susceptibility loci.^7^ However, environmental influences, especially reduced time spent outdoors, prolonged near work, and extensive screen use, are increasingly implicated in triggering earlier onset and faster progression of myopia.^8^ Medical students represent a high-risk group due to their intensive academic workload, prolonged reading hours, and high screen exposure.

Several large-scale longitudinal studies from high-income countries have consistently demonstrated a rising trend in myopia progression, particularly among adolescents and university students. In the United States, the Collaborative Longitudinal Evaluation of Ethnicity and Refractive Error (CLEERE) Study revealed that environmental exposures, including educational intensity and reduced outdoor activity, are significant contributors to the onset and progression of myopia during school years.^9^ Similarly, population-based research from the United Kingdom, such as the Avon Longitudinal Study of Parents and Children (ALSPAC), has documented increasing myopia prevalence, with rates doubling over the past five decades, particularly in urban areas.^10^

In Canada, studies led by the Canadian National Institute for the Blind have emphasized the role of screen time and near work in driving early-onset and progressive myopia among school-aged children and university students.^11^ These countries, however, benefit from advanced eye care infrastructure, widespread vision screening programs, early school-based detection, and the availability of myopia control interventions like orthokeratology, low-dose atropine therapy, and public health campaigns promoting outdoor activity.

In contrast, many low- and middle-income countries (LMICs) face substantial challenges in addressing the progression of myopia. Limited access to routine eye examinations, underdeveloped school and university vision screening programs, and insufficient public awareness often result in delayed diagnosis and inadequate treatment. Furthermore, high academic pressure, prolonged study hours, and pervasive digital screen use, particularly in urban educational settings, intensify the risk of myopia progression in young adults.^12^ Unlike high-income countries, LMICs frequently lack access to advanced myopia control strategies such as low-dose atropine therapy, orthokeratology, or public health initiatives promoting preventive behaviors. Despite the mounting burden, there remains a significant dearth of longitudinal data on myopia progression in university populations, especially those undergoing intensive academic training.^4^ Pakistan is no exception and shares these systemic limitations, highlighting the pressing need for local research and targeted interventions.

Several studies from Pakistan have investigated the prevalence and risk factors associated with myopia, particularly among students and younger populations, supporting the need for targeted surveillance and early intervention. Cross-sectional research conducted at institutions such as Khyber Medical College, Peshawar, and others has reported high prevalence rates, ranging from 50% to over 60%among medical students, with significant associations identified between myopia and behavioral factors such as prolonged screen exposure, limited outdoor activity, and academic workload.^13^ These studies also frequently highlight a strong familial predisposition to myopia within the Pakistani population. However, the existing body of literature is largely limited to cross-sectional designs, which provide only a snapshot of prevalence and associated risk factors at a single point in time. Although previous Pakistani studies have reported the prevalence and risk factors of myopia among students, most are cross-sectional in design, and longitudinal evidence on refractive progression remains limited. This lack of longitudinal data represents a major research gap and limits the formulation of targeted, evidence-based interventions for early detection and control of myopia in high-risk academic cohorts.

This research addresses this critical gap by evaluating the five-year progression of myopia among MBBS students at Fatima Jinnah Medical University, Lahore. This cohort study seeks to quantify changes in refractive status from first to final year and assess the association between study time, screen exposure, and refractive progression.

## METHODOLOGY

This study was designed as a longitudinal prospective aimed at evaluating the progression of myopia and its associated behavioral risk factors over a five-year academic period. The longitudinal design was chosen to allow longitudinal tracking of refractive changes in the same group of students for a period of five years , enabling observation of real-world trends in a naturally exposed academic population without intervention.

The research was conducted at the Department of Ophthalmology, Sir Ganga Ram Hospital, affiliated with Fatima Jinnah Medical University (FJMU) in Lahore, Pakistan. Data was collected for the MBBS student cohort admitted in 2020, covering the entire five-year duration of their academic program from January 2020 to December 2025.

### Participants:

The study population consisted of all 304 undergraduate students admitted to the MBBS program in 2020 at FJMU. Participants were included if they completed all five academic years and had complete ophthalmologic evaluations on record for each year. Students were excluded if they were detained during the study period, diagnosed with amblyopia, or had hypermetropia defined as a spherical equivalent greater than +0.50 diopters. All 304 eligible students who met the inclusion criteria were enrolled, with no loss to follow-up reported, as the data were sourced from institutional records with full retention.

### Sample Size:

The sample comprised the complete enumeration of the MBBS batch of 2020, totaling 304 students. Since the entire target cohort was followed for five years, no formal sample size calculation was required. The full coverage of the cohort strengthened the internal validity of the study findings.

### Data collection:

Data were extracted through a structured review of ophthalmology department records, student health files, and annual academic screening reports. Visual acuity was assessed annually using a standardized Snellen chart under controlled lighting conditions. Students with uncorrected vision worse than 20/20 underwent best-corrected visual acuity (BCVA) assessment using subjective and objective refraction under cycloplegia.

Refractive error was quantified in spherical equivalent refraction (SER) and recorded in diopters. Myopia progression was defined as a cumulative shift in SER of ≥ –0.50 D between the baseline assessment in 2020 and the final assessment in 2025.

Screen time and study time were documented annually through standardized interviews conducted during ophthalmologic evaluations. Students were asked to self-report their average daily hours spent on digital devices for non-academic purposes (screen time) and academic reading (study time). Where applicable, these self-reports were cross-verified with structured logs in academic wellness records to enhance accuracy. All clinical examinations were performed by trained ophthalmology residents supervised by senior consultants using consistent protocols throughout the study.

### Outcomes:

The primary outcome of the study was the progression of myopia over five years, measured as the change in spherical equivalent refraction from baseline to the final academic year. Secondary outcomes included the relationship between the degree of myopia progression and behavioral exposures, specifically daily screen time and study time.

### Statistical Analysis:

All statistical analyses were conducted using Python (version 3.10) with the SciPy and StatsModels libraries. Continuous variables such as spherical equivalent refraction (SER), daily screen time, and study time were summarized using means and standard deviations (SD) for normally distributed data, and medians with interquartile ranges (IQR) for skewed distributions. Normality was assessed using the Shapiro–Wilk test. For repeated measures across five academic years, generalized linear modeling via linear mixed-effects models (LMM) was employed to account for within-subject correlations, incorporating a random intercept for each student.

These models examined SER progression over time, with fixed effects for academic year, average screen time, average study time, gender, and family history of myopia. For longitudinal changes between Year one and Year five, multiple linear regression was applied to estimate independent associations between myopia progression (ΔSER) and covariates. Effect sizes were reported alongside 95% confidence intervals. Spearman correlation coefficients were used to explore non-parametric associations between behavioral factors and SER change. Subgroup analyses were performed using independent t-tests, Mann–Whitney U, or Kruskal–Wallis tests, where appropriate. Missing data were minimal and managed using listwise deletion. A two-tailed p-value less than 0.05 was considered statistically significant.

### Ethical considerations:

Ethical approval for this study was obtained from the Institutional Ethical Review Committee of Fatima Jinnah Medical University, Lahore, Letter no. 1122/CIERB. Informed consent was acquired from all participants at the time of their initial ophthalmologic evaluation. All data were anonymized before analysis, and participant confidentiality was maintained throughout the research process in compliance with the Declaration of Helsinki and international research ethics standards.

## RESULTS

### Descriptive statistics:

A total of 304 students were enrolled in the study, of whom 48 had complete refraction records from both Year one and Year five, allowing longitudinal estimation of spherical equivalent refraction (SER) progression. The mean baseline SER at entry (Year one) was –1.66 diopters (D) (SD ±2.41), and this progressed to –2.73 D (SD ±1.78) by the end of the five-year follow-up (Year five). The mean change in SER (ΔSER) over five years was –1.07 D (SD ±2.85), with a median progression of –0.50 D and an interquartile range (IQR) of –1.00 to 0.00 D.

Average daily screen time was 4.90 hours (SD ±1.44), while average daily study time was 3.07 hours (SD ±0.97). The range of SER progression was broad, with some students showing significant axial elongation and refractive shift (minimum ΔSER: –17.14 D), while others exhibited minimal or even hyperopic shifts (maximum ΔSER: +2.50 D) (Table-I). The distribution of SER shifted leftward from year one to year five, with a lower median and broader left tail by follow-up (Fig.1).

**Table-I:** Summary of Descriptive Statistics.

	Mean	Standard Deviation	Median	Q1	Q3	Minimum	Maximum
SER_1st	-1.66	2.41	-2	-2.69	-0.97	-7.5	7.89
SER_5th	-2.73	1.78	-2.62	-3.5	-1.44	-9.25	-0.38
ΔSER	-1.07	2.85	-0.5	-1	0	-17.14	2.5
Study Time in hrs	3.07	0.97	3	2.38	3.69	1.33	5.33
Screen Time in hrs	4.9	1.44	5	4	6	1.25	7.5

**Fig.1:**
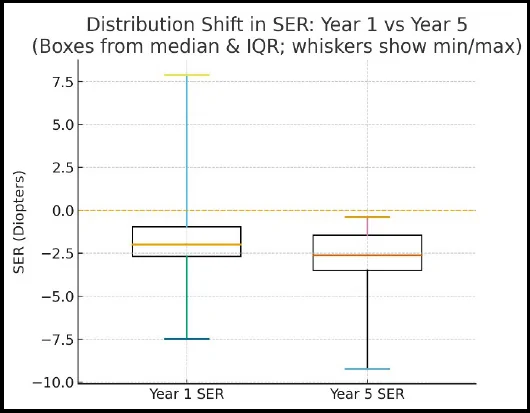
Distribution shift in spherical equivalent refraction (SER) between year one and year five. Boxes indicate the interquartile range (IQR) with median line; whiskers show observed min/max.

**Fig.2:**
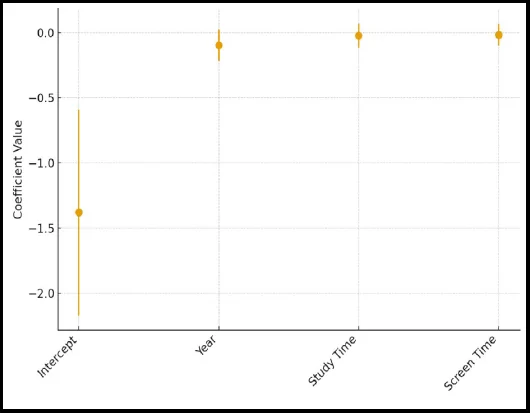
Vertical dot chart of regression coefficients.

### Normality testing:

The Shapiro–Wilk test indicated a significant deviation from normality in the distribution of ΔSER values (p < 0.00001), supporting the use of non-parametric statistical tests in subsequent correlation and subgroup analyses.

### Correlation with Behavioral variables:

Spearman’s rank correlation coefficients were used to assess associations between myopia progression (ΔSER) and behavioral exposures. No statistically significant correlation was found between ΔSER and average screen time (ρ = –0.05, p = 0.745) or study time (ρ = 0.05, p = 0.751). These findings suggest that, in this sample, screen and study durations were not independently associated with SER progression.

### Longitudinal Modeling of SER Progression:

A linear mixed-effects model (LMM) was fitted to assess the progression of spherical equivalent refraction (SER) across five academic years, accounting for within-subject correlation using a random intercept for each student (n = 304). Time was modeled as a continuous variable (Year_Num from one to five), and study time and screen time were included as covariates (Table-II).Although the average SER decreased over time (β = –0.095 D/year), this trend did not reach statistical significance (p = 0.121). Neither study time nor screen time were significantly associated with SER trajectory in this model. The large variance in the random intercept suggests considerable inter-individual variability in refractive progression.

**Table-II:** Linear Mixed Model Results for SER Progression over five Years.

Predictor	Coefficient (β)	95% CI	p-value
Intercept	–1.38	[–2.17, –0.59]	0.001
Year (1–5)	–0.095	[–0.215, 0.025]	0.121
Study Time (hrs/day)	–0.022	[–0.114, 0.070]	0.636
Screen Time (hrs/day)	–0.016	[–0.100, 0.068]	0.706
Random Intercept (SD²)	4.72	–	–

**Table-III:** Multivariable linear regression model predicting five-year myopic progression (ΔSER).

Predictor	β Coefficient	95% CI	p-value
Intercept	–1.20	[–4.02, 1.63]	0.397
Study Time (hrs/day)	–0.18	[–0.74, 0.38]	0.522
Screen Time (hrs/day)	–0.19	[–0.58, 0.21]	0.341
Baseline SER (D)	–0.96	[–1.20, –0.73]	<0.001

### Multivariable linear regression analysis of myopia progression:

To identify independent predictors of refractive error progression, a multivariable linear regression model was constructed using change in spherical equivalent refraction (ΔSER) as the dependent variable. Predictor variables included average daily study time, average daily screen time, and baseline SER at Year one. The model revealed that baseline SER was a strong and statistically significant predictor of myopia progression (β = –0.96, 95% CI: –1.20 to –0.73, p < 0.001). Neither screen time (β = –0.19, p = 0.341) nor study time (β = –0.18, p = 0.522) showed a significant association with ΔSER after adjustment. This suggests that students entering medical school with a more negative baseline refractive error were more likely to experience greater progression, regardless of behavioral exposures.

## DISCUSSION

This study provides longitudinal insight into myopia progression among undergraduate female medical students in Pakistan, contributing valuable evidence from a low- and middle-income country LMIC context. Unlike cross-sectional studies that offer a snapshot of prevalence, our five-year follow-up allowed for temporal mapping of spherical equivalent refraction (SER) changes and behavioral exposures within a high-risk academic population. The observed average myopic progression of –1.07 diopters (D) over five years underscores the public health relevance of myopia in LMIC university cohorts, especially in medically intensive environments. As academic demands rise globally, particularly in emerging economies, such data are essential to inform evidence-based myopia control strategies.

When compared to longitudinal studies in other LMICs, our findings both align with and diverge from regional trends. A study from India reported a mean progression of –0.85 D over four years among engineering students, which is slightly lower than our cohort’s –1.07 D shift, possibly due to differences in baseline SER and educational intensity.^14^ In Bangladesh, studies documented higher annual progression rates in secondary school students, yet with lower baseline refractive errors, while a recent study from Nepal highlighted progression patterns in children but lacked adolescent or university-level follow-up.^15^,^16^ Unlike these studies, our investigation focused on an adult academic cohort subjected to sustained near work and reduced outdoor exposure, providing unique insight into the burden of late adolescent myopia progression. Furthermore, Pakistan’s lack of robust national screening infrastructure or myopia control interventions distinguishes it from these neighboring countries, where community-based eye care models are somewhat more established.

Comparable findings have emerged from Southeast Asia and Sub-Saharan Africa, where rapidly shifting educational and technological landscapes have contributed to rising myopia rates. In a Malaysian study of urban university students, mean progression approached –1.00 D over three years, though with broader variability in baseline refractive status and study environments.^17^ In Nigeria, limited longitudinal data exist, but cross-sectional reports suggest underdiagnosed myopic cases due to poor access to refraction services and limited school-based screening.^18^ These differences underscore not only the importance of healthcare access but also the influence of academic culture, outdoor activity levels, and early intervention systems. In contrast to these settings, Pakistan continues to lack standardized myopia surveillance systems in universities, which may delay identification of at-risk students and limit access to preventive strategies such as optical or pharmacologic interventions. Our study, by capturing annual progression in a high-academic-load environment, helps bridge this critical evidence gap in the LMIC research landscape.

The absence of significant associations between myopia progression (ΔSER) and behavioral factors such as screen and study time was unexpected, especially given the established literature linking near work with refractive shift. Several factors may explain this discrepancy. First, the strong predictive power of baseline SER in our regression models suggests a possible ceiling effect, individuals entering with higher baseline myopia were more likely to progress, regardless of behavior.^19^ This aligns with hypotheses that axial elongation may follow a predetermined trajectory once initiated, particularly in genetically predisposed individuals.^20^ Second, the retrospective, self-reported nature of screen and study time introduces potential recall bias and measurement imprecision, possibly diluting true associations. Finally, homogeneity in exposure, nearly all students reported high academic and screen engagement, may have limited the ability to detect significant differences, given the narrow variance in these predictors.

### Limitations

The single-center, all-female sample restricts generalizability to broader student populations, including males and non-medical disciplines. This attrition likely reflects systemic gaps in long-term vision monitoring within academic institutions rather than participant noncompliance, reinforcing the need for structured screening programs.

## CONCLUSION

This five-year longitudinal cohort study provides critical evidence on the progression of myopia among female medical students in Pakistan, highlighting a substantial average refractive shift of –1.07 D during the academic period. The strong predictive role of baseline refractive status, rather than modifiable behavioral exposures, underscores the importance of early identification and risk stratification in high-academic-load populations. Given the absence of national screening frameworks and the growing burden of myopia in low- and middle-income countries, our findings call for urgent implementation of structured surveillance, early intervention strategies, and longitudinal monitoring to mitigate long-term visual morbidity in at-risk student cohorts.

### Future recommendations:

Future research should prioritize multi-institutional prospective designs involving larger and more diverse populations across urban and rural settings in Pakistan. Incorporating objective measures of near work and light exposure, such as wearable activity monitors or digital tracking software, would improve precision. Moreover, axial length measurements should be integrated into future protocols to differentiate true structural elongation from refractive compensation. There is also a need to evaluate the feasibility and effectiveness of low-dose atropine, optical interventions (e.g., defocus lenses), and public awareness campaigns in resource-limited environments. Ultimately, establishing national guidelines and vision screening protocols for university students may mitigate the overlooked burden of progressive myopia in LMICs.
